# High-efficiency grating-couplers: demonstration of a new design strategy

**DOI:** 10.1038/s41598-017-16505-z

**Published:** 2017-11-30

**Authors:** Riccardo Marchetti, Cosimo Lacava, Ali Khokhar, Xia Chen, Ilaria Cristiani, David J. Richardson, Graham T. Reed, Periklis Petropoulos, Paolo Minzioni

**Affiliations:** 1 University of Pavia, Department of Electrical Engineering and Computer Science, Pavia, IT27100 Italy; 2 University of Southampton, Optoelectronics Research Centre, Southampton, SO17 1BJ United Kingdom

## Abstract

We present a simple and practical strategy that allows to design high-efficiency grating couplers. The technique is based on the simultaneous apodization of two structural parameters: the grating period and the fill-factor, along with the optimization of the grating coupler etching depth. Considering a 260 nm Si-thick Silicon-on-insulator platform, we numerically demonstrated a coupling efficiency of −0.8 dB (83%), well matching the experimental value of −0.9 dB (81%). Thanks to the optimized design, these results represent the best performance ever reported in the literature for SOI structures without the use of any back-reflector.

## Introduction

In recent years silicon photonics has established itself as a mature technology for the development of integrated and low cost optical devices for telecom applications^1^. Various optical components such as modulators^2^, optical filters^3,4^, photodetectors^5^, arrayed waveguide gratings (AWGs)^6^, and all-optical wavelength converters^7^ have successfully been demonstrated, showing that multiple functions can be effectively integrated in a single integrated photonic chip^8^. Considering Silicon-on-Insulator (SOI) technology, the strong refractive index difference between Si and SiO_2_ allows the implementation of very small, single mode waveguide structures^9^. On one hand this enables dense on-chip device integration, but on the other side it makes efficient fiber-to-waveguide coupling particularly challenging because of the vast difference in the mode areas (≈80 *μm* ^2^ for fibers and <0.2 *μm* ^2^ for SOI waveguides). Among the various proposed solutions, two different approaches have attracted most attention: edge-coupling and grating-coupling. By exploiting edge-coupling, insertion losses lower than 0.5 dB can be achieved over a broad bandwidth (>100 nm)^10^. Nevertheless, this technique requires complicated post-fabrication processes, such as high quality facet polishing, and high-resolution optical alignment, making it not suitable for wafer-level testing and high-volume manufacturing. Grating couplers (GC), on the other hand, represent a very attractive solution, since they can be placed anywhere on the chip, allowing simple wafer-scale automated testing^1^ and providing much wider alignment tolerance than that allowed by edge-coupling. The main drawbacks of standard uniform GC are their narrow bandwidth (usually between 30–40 nm, less than half of edge-coupling solutions) and the relatively low coupling efficiency (CE), which is usually lower than 61%^11^. The relatively low CE can be attributed to two main factors: grating directionality and mode mismatch. Considering a beam coupling from the SOI waveguide to a fiber, a low grating directionality (≈65%) means that a large percentage of the optical power incident on the GC is diffracted towards the substrate (≈35%) instead of being sent towards the fiber. The mode mismatch limitation is due to the fact that the optical intensity profile radiated by a uniform grating shows an almost exponential-decay shape with a reduced overlap integral with the mode profile (almost Gaussian) of standard optical fibers^11^. Different solutions have been proposed in the literature to increase the directionality by reducing the power leakage to the substrate. They make use of different approaches, such as the use of either poly-Silicon over-layers^12,13^ or back-reflectors embedded in the substrate, e.g. distributed Bragg reflectors (DBRs)^11,14^ and metallic mirrors^15–17^. All of these techniques enhance the GC directionality, but require additional fabrication steps, that sometimes even involve the use of non-CMOS-compatible materials. CE can also be increased by tailoring the amount of optical power scattered by each element of the grating, so as to reduce the mode mismatch between the radiated field profile and the optical mode in a single-mode fiber (SMF). This is generally achieved by varying either the etching depth^18^ or the fill-factor of each grating period^11,12,16,17,19–22^. Alternatively, the hole-size in fully-etched photonic crystals (PhC) may be varied along the grating, however this often requires more complex fabrication processes^17,23^. Apodized GC are generally designed by applying numerical techniques, such as genetic algorithms (GA), to optimize a simple starting-structure. These techniques, in addition to being computationally intensive and time-consuming, do not allow getting a real physical insight on why a particular apodization curve yields to a CE increase. In this paper, we start by discussing the physical principle underlying the design of apodized GC with optimal CE in Section 2 and then describe the fabrication procedure and experimental characterization results in Section 3. Counter-intuitively, the obtained GC achieves higher CE than GA-optimized designs reported in the literature that do not use back-reflectors and the reason for this will be clear by the end of the paper.

## Grating design and simulation

**Figure 1:**
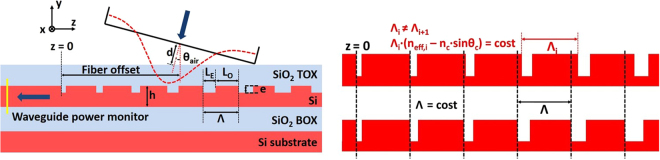
Left: Cross-sectional schematic and simulation layout of a non-uniform GC in a SOI wafer, based on a linear apodization of the grating *F*. Right: Cross-sectional schematic of the proposed linearly apodized GC (top) compared to that of a linearly apodized grating with fixed period Λ (bottom).

In standard SOI uniform GC, trenches having a length *L*_*E*_ and depth *e* are etched in the silicon layer with a periodicity Λ. If we define the grating fill-factor *F* as the ratio between the length of the un-etched section *L*_*O*_ to the total length Λ of the radiative unit (see Fig. 1(left)), we can express the effective index of the grating *n*_*eff*_ as

1 $${n}_{eff}=F\cdot {n}_{O}+\mathrm{(1}-F)\cdot {n}_{E}$$

where *n*_*O*_ and *n*_*E*_ are the effective indices of the original silicon slab and the etched areas, respectively. The periodic refractive index change between the trenches and the un-etched teeth, allows the optical mode propagating in the silicon waveguide to be diffracted to free space. According to the first order Bragg condition, the grating periodicity Λ is given by

2 $${\rm{\Lambda }}=\frac{{\lambda }_{c}}{({n}_{eff}-\,\sin \,{\theta }_{air})}$$

where *λ*_*c*_ is the coupling-wavelength, *θ*_*air*_ is the diffraction angle, and *n*_*eff*_ is the effective refractive index of the radiative unit, as shown in Fig. 1(left). Starting from the basic structure of a uniform GC, we decided to introduce a linear apodization on the grating to improve the CE. It is well known that by linearly varying the *F* value along the grating, two positive effects can be achieved: the amount of optical power radiated by the first elements of the grating is reduced, and the optical impedance matching between the waveguide and the grating section is improved^20^.

The equation used to apodize the grating can then be expressed as

3 $$F={F}_{0}-R\cdot z$$

where *F*_0_ is the initial fill-factor of the first radiative unit, *R* is the linear apodization factor and *z* is the distance of each radiative unit from the starting point of the grating. A cross-sectional schematic of the grating is shown in Fig. 1(left). It is important to highlight that Eq. 3 describes the evolution of *F* along the grating, but does not impose any limitation on Λ, which still remains a free parameter. In the majority of the works reported in the literature that discuss linearly-apodized GC, Λ is considered to be constant along the whole grating length^20–22^. This assumption prevents from simultaneously satisfying the Bragg condition by all of the grating elements: as *F* is varied along the grating, the effective index *n*_*eff*_ of each radiative unit changes, and thus the Bragg condition is satisfied only at a specific point of the grating but not along the whole structure. To alleviate this, we decided to design a GC with a linear apodization of *F* and a value of Λ that was varied along the structure, so as to satisfy the Bragg condition along the whole grating. To calculate the effective index *n*_*eff*_ of each radiative unit, according to the chosen level of etch depth *e*, Eq. 1 was used. The values of *n*_*O*_ and *n*_*E*_ were extracted from mode simulations of slab waveguides having height respectively equal to *h* and *h* − *e*, these two quantities representing the thickness of the Si layer of the wafer, for the non-etched (teeth) and etched (trenches) areas respectively. Consequently, while *n*_*O*_ is a constant value, *n*_*E*_ depends on the etch-depth *e*, and consequently also *n*_*eff*_ is a function of *e*. Using the obtained values of *n*_*eff*_ and taking advantage of Eq. 2 we calculated the length of the teeth (*L*_*O*_) and trenches (*L*_*E*_) of each radiative unit to optimize the CE for a beam with *λ* = 1550 nm and an angle of incidence *θ*_*air*_ of 14.5°. A graphical representation of the difference between our linear apodization scheme and the standard approach based on fixed Λ, is given in Fig. 1(right). It is worth noting that using the proposed approach, and considering the standard situation where the thickness of the Si layer (*h*) and BOX layers are imposed by the available SOI wafers, only two design parameters need to be optimized: the etch depth *e* and the apodization factor *R*; this is rather different from the case of GA-optimized techniques, which specify the lengths of the etched and un-etched portions of each radiative unit. Concerning the choice of the initial fill-factor *F*_0_ in our design, it has been shown that reducing *F*_0_ results in an increase in the grating CE^22^. Unfortunately, the value producing the highest CE (*F*_0_ = 0.95^22^) implies a minimum feature size of about 60 nm, which is not achievable with fabrication processes available to us. We therefore set *F*_0_ to 0.9, which corresponded to a value of *L*_*E*_ = 60 nm for the first trench. Full vectorial 2D-FDTD simulations using FDTD Solutions^TM^ (from Lumerical Inc.) were carried out in order to find the optimum values of the *e* and *R* parameters for the structure shown in Fig. 1(right). The details of the numerical simulations are reported in the Methods section. We took into consideration two different types of SOI wafer, both having a 2-*μ*m-thick buried SiO_2_ layer (BOX, ${n}_{ox}=1.44$ at 1550 nm) and having a Si-layer (${n}_{Si}=3.48$ at 1550 nm) of height $h=220\,nm$ and $h=260\,nm$ respectively. A top SiO_2_ layer (TOX, ${n}_{ox}=1.44$) was included in the layout, having a height of 720 nm for the 220 nm SOI wafer, and 680 nm for the 260 nm SOI wafer. Trenches were assumed to be completely filled by the TOX and no index-matching liquid was considered between the TOX and the fiber.

**Figure 2:**
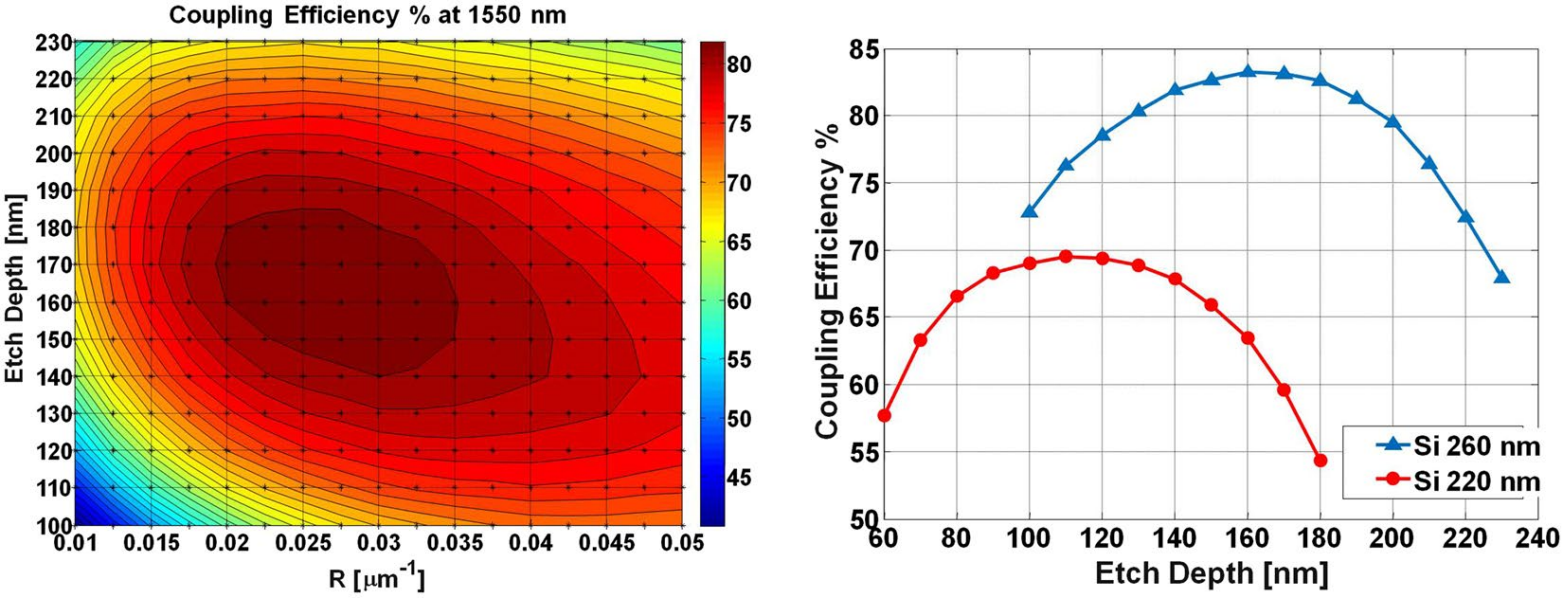
Left: Contour plot of the peak CE at *λ* = 1550 nm of the linearly apodized GC realized in 260 nm SOI platform, as a function of etch depth and of the linear apodization factor *R*. Right: maximum CE at the central wavelength of 1550 nm for the linearly apodized GC based on 220 nm SOI (red plot) and 260 nm (blue plot).

We evaluated the CE by considering the grating as an in-coupling device, i.e. coupling light from a SMF into the SOI waveguide by means of the GC. The fiber, which was assumed to have an outer diameter of 125 *μ*m and a mode field diameter (MFD) of 10.4 *μ*m, was tilted by *θ*_*air*_ = 14.5° with respect to the vertical direction (corresponding to an incidence angle on the grating of *θ*_*c*_ = 10° in the TOX), causing the Gaussian beam output from the fiber to impinge on the TOX with a MFD of about 10.8 *μ*m. The selected angle of incidence helped in reducing back-reflections into the launch-fibre and, at the same time, provided directionality to the light coupled into the waveguide taper. The electric field of the Gaussian beam was polarized along the $\hat{{\rm{x}}}$ direction with reference to Fig. 1(left), so that the incoming light could be coupled to the fundamental TE mode of the integrated waveguide. A frequency-domain power monitor, positioned along the Si waveguide at a 10 *μ*m distance from the grating, was used to measure the amount of optical power coupled to the waveguide. The CE of different grating configurations was calculated while simultaneously sweeping both the etch depth *e*, by 10 nm steps, and the apodization factor *R*, by 0.0025 *μ*m^−1^ steps. For every grating configuration, the offset of the fiber mode from the starting point of the grating was also optimized. To illustrate an example of the simulation results, we plot in Fig. 2(left) a contour diagram showing the peak CE at 1550 nm for a 260-nm thick Si-layer as a function of the parameters *e* and *R*. It can be noticed that, starting from an etch depth of 100 nm, the maximum CE is found to be 73% (−1.4 dB) when the apodization factor *R* is equal to 0.0425 *μ*m^−1^.

Increasing the etch depth while reducing the factor *R*, yields a large CE improvement, reaching a maximum of 83% (−0.8 dB) for *e* = 160 nm and *R* = 0.025 *μ*m^−1^. If the etch depth is further increased, the CE starts to decrease, with a value of 68% (−1.7 dB) when *e* = 230 nm and *R* = 0.025 *μ*m^−1^. It is also interesting to note that the simulated CE is higher than 80% for a wide range of (*e*, *R*) combinations, thus showing a good tolerance to small variations in the fabrication process. The same procedure used for the design of the apodized GC based on the 260 nm SOI platform, was also applied to the 220 nm SOI grating, achieving a maximum CE of 70% (−1.6 dB) when *e* = 110 nm and *R* = 0.0275 *μ*m^−1^. The maximum CE achieved at the central wavelength of 1550 nm, as a function of the etch-depth, for each of the two SOI platforms is reported in Fig. 2(right). It can be seen that by using a SOI platform with a thicker Si layer it is possible to obtain a higher CE, as already reported in other works^20^, and that deep etching levels (110 nm and 160 nm for the 220 nm and 260 nm SOI platforms, respectively) are required to obtain the maximum CE.

**Figure 3:**
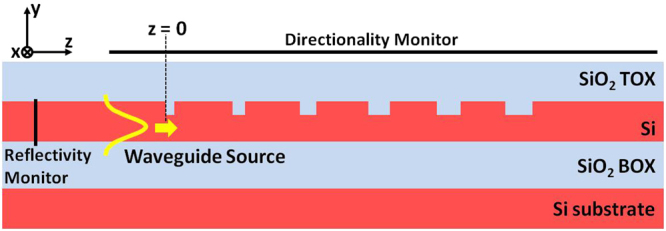
Cross-sectional schematic and simulation layout used to assess the directionality and reflectivity of uniforms and apodized GC.

To complete the GC analysis we also simulated the grating as an out-coupling device. We set a fundamental-mode waveguide source in the Si waveguide, a power-monitor above the GC to assess its directionality and an additional power-monitor in the Si-layer to assess grating reflectivity by measuring the optical power reflected back in the waveguide, as shown in Fig. 3.

**Figure 4:**
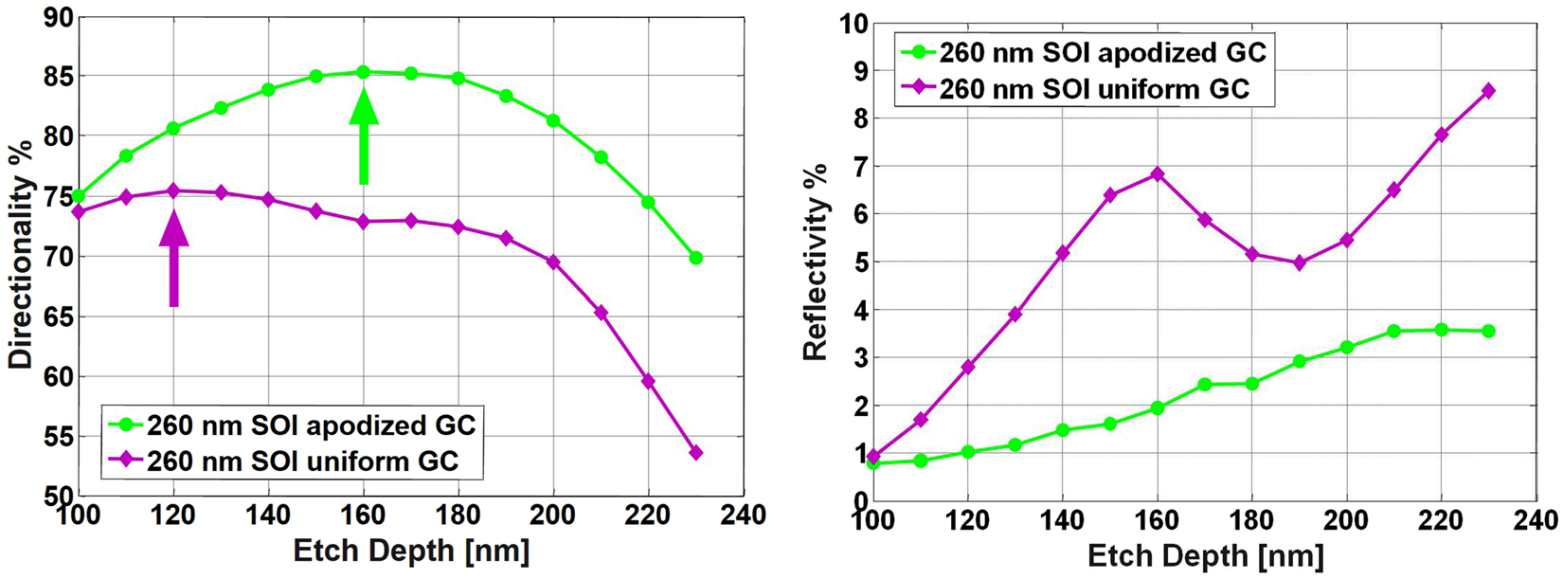
GC directionality (left) and reflectivity (right) at *λ* = 1550 nm as a function of *e* for the proposed linearly-apodized GC (green curve) and for a uniform GC (purple curve). The arrows in the left panel indicate the maximum directionality achievable in each structure.

The analysis was carried out for the 260 nm Si-thick SOI platform, comparing the performances of our apodized design at *λ* = 1550 nm to that of uniform gratings (*F* = 0.5) realized in the same platform and having the same TOX height. Directionality results are shown, as a function of etch depth *e*, in Fig. 4(left). It can be noted from the figure that the maximum directionality achievable by the apodized GC is equal to 85%, almost 10% better than the maximum directionality achievable with the uniform design. Since TOX and BOX values were kept constant, this shows that directionality is significantly improved by the adoption of the proposed apodization technique. It is also worth noting, that the optimum value of the etching depth is different when a uniform GC is considered rather than the apodized one (see Fig. 4(left)). This demonstrates that, in order to design the optimum GC, both of the apodization profile and the etching depth must be simultaneously optimized. This aspect was not considered in previous works such as in^19^, leading to a reduced directionality and CE.

We report the reflectivity for both uniform and apodized GC as a function of the etch-depth in Fig. 4(right). The proposed design strategy allows a significant reduction in the GC reflectivity with respect to the uniform structure. Moreover, it also suggests that the big difference in the identification of the optimal etch-depth between uniform and apodized GC could be related to the large reflectivity increase observed in the uniform GC around 160 nm.

A comparison of our result to those obtained in previous works (see Table 1) shows that our design strategy allowed for the first time a CE better than −1 dB (considering a Si thickness <340 nm and without using a back-reflector). A direct comparison with^21^ and^22^, in which constant-Λ linear-apodization gratings were employed, shows that our design approach achieves a CE improvement of at least 1 dB. Furthermore, our results also show a non-negligible CE improvement with respect to results reported in^20^, where a constant-Λ linear-apodization design was used as the starting configuration for a GA based refinement, thus highlighting the importance of allowing Λ to vary.

**Table 1:** Summary of the theoretical (CE_T_) and experimental (CE_E_) coupling efficiencies for different GC reported in the literature. Results are ordered with respect to the thickness of the Silicon layer. Devices that required the use of a back reflector (BR) are reported in italics, while those relating to this work are shown in bold. All the values are reported with the number of significant figures provided by the authors.

Si [nm]	Description	CE_T_ [dB]	CE_E_ [dB]	Ref.	Si [nm]	Description	CE_T_ [dB]	CE_E_ [dB]	Ref.
220	GA	−2.15	—	11	250	fully-etched PhC	−1.8	−1.74	23
220	poly-Si overlay	−1.08	—	12	250	lag effect in etching	−1.31	−1.9	18
220	poly-Si overlay	—	−1.6	13	250	linear apodization	−2.3	−2.7	22
220	linear apodization	−2.6	−2.7	21	250	BR: Aluminum	−0.26	−0.62	16
220	GA	−1.9	—	20	250	BR: Aluminum	−0.43	−0.58	17
220	BR: Gold	−1.43	−1.61	15	260	GA	−1.0	—	20
220	BR: DBR	−0.36	—	11	260	linear apodization	−0.8	−0.9	*
220	BR: DBR	−0.86	−1.58	14	340	200-nm deep etching	−0.8	−1.2	19
220	linear apodization	−1.6	—	*	340	GA	−0.5	—	20

We also applied our design procedure to the case of a SOI wafer with 340 nm-thickness Si-layer (as in^19^), obtaining a CE of 85% (−0.7 dB), for *e* = 210 nm and *R* = 0.0425. The obtained CE is slightly higher than that reported in^19^ where a fill-factor (*F*) apodization suitable to produce a Gaussian-shaped beam profile (CE = 83%; −0.8 dB) was considered. The improvement achieved using the proposed design strategy can be explained by considering two aspects: i) only minor differences are present between the *F* apodization curve reported in^19^ and a linear apodization and ii) identifying the optimal etch depth and the single-element scattering coefficient in uniform GC does not guarantee obtaining the optimal CE, as previously shown (see Fig. 4). In comparison to results obtained by application of GA, it is important to stress that GA generally require a significant computational effort. For this reason different parameters (such as etch-depth or the grating period) are generally considered as fixed, thus allowing to reduce the computation time but, simultaneously, limiting the explored solutions-range. Using the proposed design approach, we limited the explored design-set to linearly apodized GC, focusing our optimization efforts on three parameters, i.e. period (thanks to Eq. 2), etch depth and fill-factor apodization, which significantly affect the GC-CE, as discussed above. This eventually led to the optimal linearly apodized GC design of this work.

## Results

**Figure 5:**
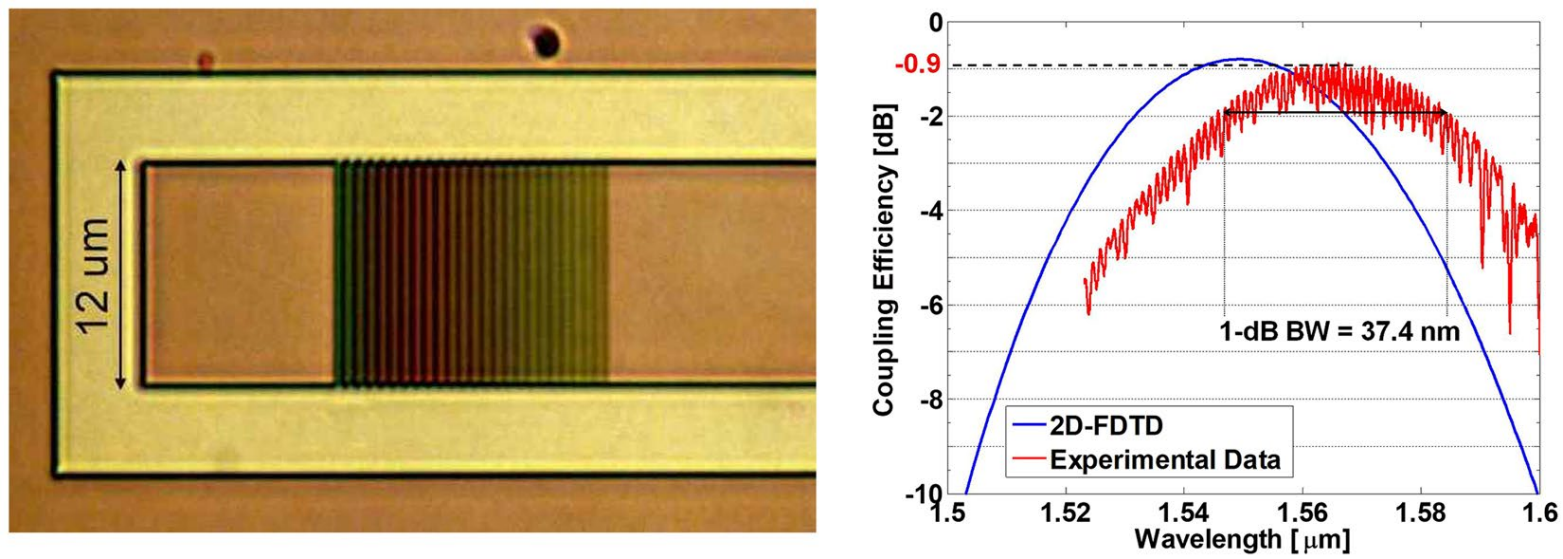
Left: Optical micrograph of one of the apodized GC. Right: CE of the best-performing grating (red trace) together with the simulated CE.

We then fabricated and characterized (see Methods section) the grating-coupler structure identified as optimal by the previous analysis. One of the measured CE curves as a function of wavelength is shown in Fig. 5(right), together with the 2D-FDTD simulation result.

**Figure 6:**
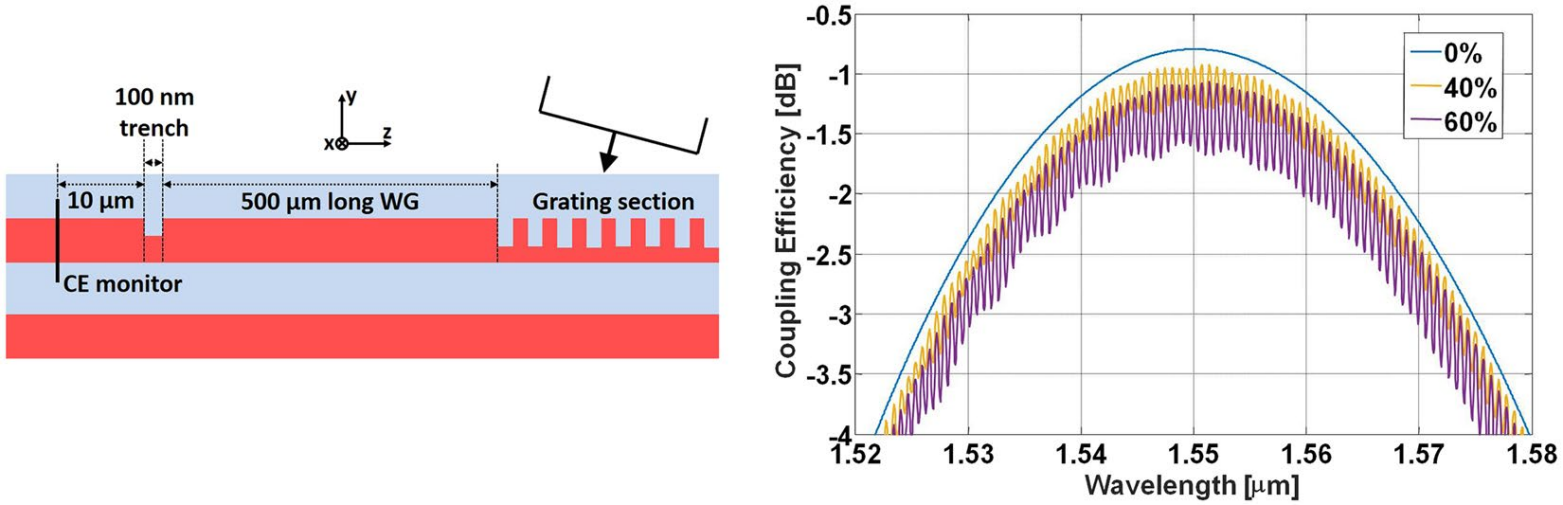
Left: Cross-sectional schematic and simulation layout (not in scale) used to evaluate the impact of a back-reflecting element (the 100-nm wide trench) on the measured CE. Right: 2D-FDTD simulation of the optimum grating transmission, when a 100 nm wide hole is inserted in the Si waveguide at a distance of 500 *μ*m from the end of the grating section, at different etching percentage. The blue curve represents the unperturbed case, while the yellow and purple curves correspond to hole etching percentages of respectively 40% and 60%.

We believe that the parasitic amplitude ripple affecting the grating transmission spectrum is caused by the transition between the taper and the single-mode waveguide. The period of the ripple is indeed compatible with a cavity having the same length as the taper section. We carried out specific simulations in order to ensure that the interaction between the non-ideal taper and the grating would not lead to an unexpected spectral redistribution of the transmission efficiency. It is in fact important to clarify if the CE achievable with a proper taper would correspond to the maximum or the average values of the observed ripple. To investigate this aspect, we performed 2D-FDTD simulations of the apodized GC, inserting a 100 nm wide trench in the Si waveguide, at a distance of 500 *μ*m from the end of the grating section. The transmitted optical power was assessed by a frequency-domain power monitor, positioned along the Si waveguide 10 *μ*m after the trench. By varying the trench depth we simulated different amounts of back-reflection at the taper-waveguide interface, and obtained the results reported in Fig. 6: the blue, yellow and purple curves show the transmission function obtained by considering an etch-depth of the trench equal to 0%, 40% and 60% of the Si-layer, respectively. As expected, increasing the etch-depth leads to an increasing amplitude of the ripple and to a reduction of the maximum CE. It is interesting to notice that the peaks of the ripple never exceed the threshold set by the CE of the unperturbed grating (0% etch, blue line), meaning that the maximum CE of the analyzed sample can be conveniently taken to correspond to one of the maxima of the parasitic oscillation.

We performed measurements on several different structures, from four different chips, obtaining an average CE of −1.1 dB, and a maximum CE of −0.9 dB. A summary of the measured coupling efficiencies for all of the analyzed devices is given in Table 2. These results show a very good agreement with the theoretical CE values (−0.8 dB, i.e. 83%), and indicate a good consistency in the fabrication process. The discrepancy between the simulated −1 dB bandwidth of 32.8 nm and the experimentally derived one, with an average value of 38.8 nm and a maximum value of 39.8, can be explained by small fabrication imperfections and variations over the wafer, such as variations in the actual etch depth or in the uniformity of the deposited TOX layer.

**Table 2:** Summary of the fabricated gratings coupling efficiency and −1 dB bandwidth, and waveguide propagation loss, for all of the measured chips.

Struct.	Chip 1		Chip 2		Chip 3		Chip 4	
	C.E. [dB]	BW. [nm]	C.E. [dB]	BW. [nm]	C.E. [dB]	BW. [nm]	C.E. [dB]	BW. [nm]
1	1.0	38.4	1.0	39.8	1.4	37.8	1.2	39.4
2	1.0	38.8	0.9	37.4	1.4	39.8	1.3	39.3

It is worth highlighting that, as reported in Table 1, the experimentally measured CE is better than that reported in^21^ and^22^ (−2.7 dB), where a linear grating apodization was used, and even better than the value (−1.2 dB) obtained by using a fill-factor apodization in a 340 nm Si-thick SOI wafer^19^. The relatively large deviation between theoretical and experimental data in^19^ can probably be associated to the high aspect ratio of the required trenches (>4.5 compared to 2.7 in the proposed structure), whose proper fabrication can be quite challenging.

Our result is also quite close the record value of −0.62 dB reported in^16^, which was achieved using an Al back-reflector in a 250 nm Si-thick SOI wafer, thus proving that that a very high CE, below the −1 dB threshold, can be obtained even without the use of any back-reflector.

## Conclusion

In this paper, we described a new design strategy for apodized diffractive gratings yielding highly efficient optical coupling between SOI waveguides and SMF. Thanks to a simultaneous apodization of both the grating fill-factor and the period along the optimization of the GC etching depth, we demonstrated a theoretical CE up to 70% (−1.6 dB) when using a 220 nm Si-thick SOI platform, and up to 83% (−0.8 dB) when a using a 260 nm Si-thick SOI platform. Experimental characterization of different samples, fabricated on a 260-nm Si-thick SOI wafer by E-beam lithography and a single-etch process, showed that thanks to the optimized design, an average CE of −1.1 dB and average −1 dB bandwidth of 38.8 nm were achieved. The highest CE experimentally measured was −0.9 dB, which currently represents the best result ever obtained without the use of embedded back-reflectors.

## Methods

### Numerical Simulations

Full vectorial 2D-FDTD simulations were carried out using FDTD Solutions^TM^ (by Lumerical Inc.). The 2D computational area was set to be 33.4 *μ*m wide and 5.8 *μ*m high, while the refractive index of both Silicon and SiO_2_ were calculated following the data reported by Palik: as a consequence the refractive indices at the design wavelength of 1550 nm are ${n}_{Si}$ = 3.48 and ${n}_{Si{O}_{2}}$ = 1.44. The simulation grid was defined using the conformal mesh method embedded in the software and setting the mesh accuracy to 8 (the highest possible value), which corresponds to a mesh grid minimum feature of 12 nm inside the material. Each simulation required, on a Intel(R) Core(TM) i7-3930K CPU (@3.20 GHz) computer, about 216 MB of RAM (initialization and mesh: 51 MB, simulation running: 72 MB, data collection: 93 MB) and a computation time of about 50 seconds. The diffracted power was calculated using the frequency-domain power monitors, set with 500 frequency points from a minimum wavelength of 1.425 *μ*m to a maximum wavelength of 1.675 *μ*m.

### Sample Fabrication

Based on simulation results, we fabricated the apodized GC design yielding the highest CE in a 260 nm Si-thick SOI platform (*e* = 160 nm, *F*_0_ = 0.9, *R* = 0.025 *μ*m^−1^). The grating was 12 *μ*m wide (along the $\hat{{\rm{x}}}$ direction of Fig. 1(left)), to properly accommodate the Gaussian mode of the fiber, and 14.847 *μ*m long. The length of each tooth (*L*_*O*_) and trench (*L*_*E*_) of the grating is given in Table 3. To assess the grating CEs’ we designed structures composed of two GCs’, connected by a straight single-mode waveguide, having width equal to 500 nm, and length equal to 5.2 mm. An additional set of 5 spiral waveguides (with lengths ranging from 6.2 mm to 26.2 mm, at 5 mm steps) was designed, so as to allow separating the contributions of grating coupling and waveguide propagation losses. The waveguides were connected to the gratings by using 500 *μ*m long linear tapers. Electron Beam Lithography (E-beam) was used to define the sample pattern and Inductively-coupled plasma (ICP) dry etching defined the final structures.

**Table 3:** Optimal trench and tooth width obtained from the optimization of the apodized GC in 260 nm SOI platform, having *F*_0_ = 0.9 and *R* = 0.025 *μ*m^−1^.

Period	*L*_*E*_ (nm)	*L*_*O*_ (nm)	Period	*L*_*E*_ (nm)	*L*_*O*_ (nm)	Period	*L*_*E*_ (nm)	*L*_*O*_ (nm)
1	60	540	9	138	484	17	225	422
2	69	533	10	148	477	18	237	413
3	79	526	11	159	469	19	249	405
4	88	520	12	170	461	20	261	396
5	98	513	13	180	454	21	273	387
6	108	506	14	191	446	22	286	378
7	118	498	15	203	438	23	298	369
8	128	491	16	214	430	24	311	//

A protective TOX layer was finally deposited by means of plasma enhanced chemical vapor deposition (PECVD). An optical micrograph of the fabricated apodized GC is shown in Fig. 5(left).

### Experimental Characterization

The experimental characterization was carried out by means of a vertical coupling scheme, using polarization maintaining (PM) single mode fibers. The optical source was a PM external cavity laser (ECL), tunable from 1523 to 1600 nm at 5 pm steps, and a power meter was used to measure the output optical power collected from the test structures. The grating CE was obtained by subtracting the waveguide propagation loss (measured on the spiral structures) from the measured fiber-to-fiber transmission and dividing by two (thus assuming that the input and output coupling losses were equal). Propagation losses were independently measured across the four samples and were assessed to be ${\alpha }_{S1}=3.1\pm 0.12$ dB/cm, ${\alpha }_{S2}=3.3\pm 0.12$ dB/cm, ${\alpha }_{S3}=3\pm 0.12$ dB/cm and ${\alpha }_{S4}=4.5\pm 0.12$ dB/cm.
